# The Roles of the PRDM Family in the Neuro-Motor System

**DOI:** 10.3390/biom16040497

**Published:** 2026-03-26

**Authors:** Shiqi Deng, Hui Li, Jie Feng, Jun Zou, Lingli Zhang

**Affiliations:** 1 College of Athletic Performance, Shanghai University of Sport, Shanghai 200438, China; 2421811003@sus.edu.cn; 2 School of Exercise and Health, Shanghai University of Sport, Shanghai 200438, China; lihui@suat-sz.edu.cn (H.L.); 2321517006@sus.edu.cn (J.F.); 3 Chinese Academy of Sciences, Shenzhen University of Advanced Technology, Shenzhen 518055, China

**Keywords:** PRDMs, physiological system, diseases, metabolic homeostasis

## Abstract

The PRDM (PR domain-containing) family consists of transcriptional regulators characterized by a PR (PRDI-BF1 and RIZ homology) domain, a subtype of the SET domain, and a variable number of zinc finger motifs. Nineteen PRDM family members have been identified in both mice and humans, and increasing evidence supports their roles as epigenetic regulators in development and disease. PRDM proteins share a conserved structure, comprising an N-terminal PR domain with potential histone methyltransferase activity and C-terminal C2H2-type zinc fingers involved in protein–protein, protein–RNA, and protein–DNA interactions. Recent studies indicate that multiple PRDM family members are involved in the regulation of the neuro-motor system, including neural lineage specification, neuronal differentiation, motor function maintenance, and neuromuscular-related pathological processes. This review summarizes current evidence on the functions and regulatory mechanisms of PRDM family members in the neuro-motor system. Overall, PRDM family members act as important epigenetic regulators in the neuro-motor system. Clarifying their molecular mechanisms may contribute to a better understanding of neuro-motor regulation and provide a theoretical basis for future research in exercise and movement science.

## 1. Introduction

The PRDM (PR domain-containing, PRDI-BF1, and RIZ homology domain) protein family first emerged in metazoans, subsequently expanded in vertebrates, and underwent further duplication events in primates [1]. PRDM proteins constitute a group of transcriptional regulators characterized by a conserved structural organization, including an N-terminal PR (PRDI-BF1 and RIZ homology) domain and a variable number of C2H2-type zinc finger motifs at the C-terminus. The PR domain is associated with potential histone methyltransferase activity, whereas the zinc finger domains mediate interactions with DNA, RNA, and other proteins. Notably, some members, such as PRDM11 and specific splice variants of PRDM7, lack canonical zinc finger motifs [1,2]. In addition, most PRDM genes generate two major isoforms through alternative promoter usage or splicing: a PR domain-containing (PR-positive) form and a PR domain-lacking (PR-negative) form, which are often associated with distinct or even opposing biological functions. These isoforms have been extensively implicated in tumorigenesis through diverse mechanisms, including mutation, deletion, and epigenetic dysregulation [3,4,5].

Coordinated movement is a complex biological process that depends on the integrated function of multiple systems, including the nervous system, skeletal muscle, and the skeletal framework. Neural circuits regulate motor neuron activity and signal transmission; skeletal muscles generate force; and bones provide structural support and mechanical stability. These components operate as an interconnected functional unit, in which precise coordination is essential for maintaining motor performance and physiological homeostasis. Accordingly, the neuro-motor system can be understood in an integrative sense that encompasses neural, muscular, and skeletal components. Emerging evidence links PRDM family members to the neuro-motor system; however, current knowledge is uneven, with only a subset (e.g., PRDM1, PRDM12, PRDM13, and PRDM16) well characterized, while many others remain poorly understood.

**Figure 1:**
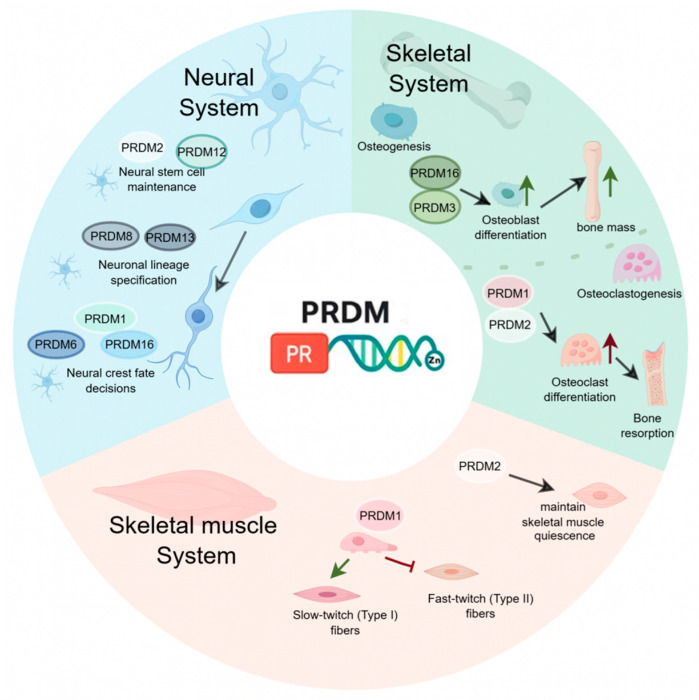
Integrated roles of the PRDM family in the neuro-musculoskeletal system. This figure summarizes the multifaceted functions of PRDM family members across the neural, skeletal, and skeletal muscle systems. In the neural system, PRDM proteins regulate key neurodevelopmental processes, including neural stem cell maintenance, neuronal lineage specification, and neural crest cell fate determination, thereby shaping neural identity and circuit formation. Within the skeletal system, PRDM factors maintain bone homeostasis by regulating osteoblast differentiation and osteoclastogenesis, thereby controlling bone formation and resorption dynamics. In the skeletal muscle system, PRDM members contribute to muscle stem cell (satellite cell) quiescence, coordinate myogenic differentiation, and regulate muscle fiber-type specification, ultimately supporting muscle maintenance and functional specialization.

**Figure 2:**
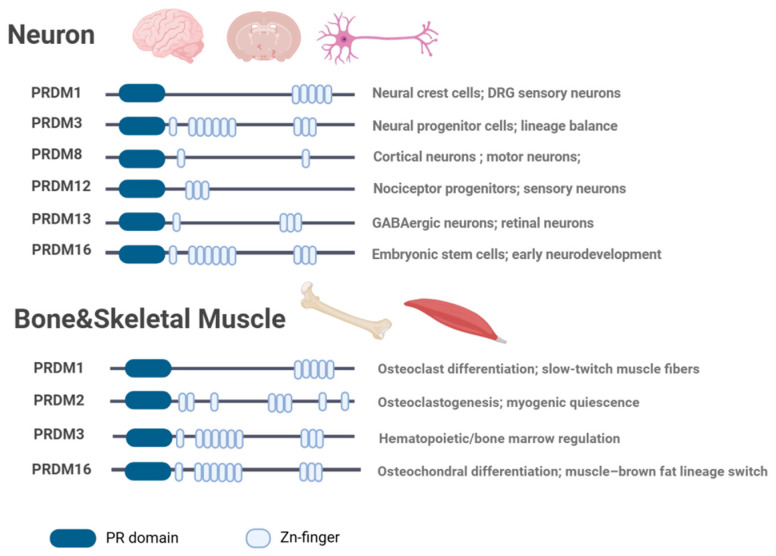
Expression patterns and domain organization of representative PRDM family members in the neuro-musculoskeletal system. This figure summarizes the domain organization of PRDM proteins and their roles in the nervous and skeletal systems. In the nervous system, PRDM members regulate neural lineage specification, neuronal differentiation, and stem cell maintenance. In the skeletal system (bone and skeletal muscle), they are involved in osteoclast differentiation, osteochondral development, myogenic differentiation, and maintenance of myogenic quiescence. Representative members are shown based on current evidence.

In this review, we summarize the structural and functional characteristics of PRDM family members and discuss recent advances in their roles across the neuro-motor system (Figure 1 and Figure 2). By adopting an integrative perspective that includes neural, muscular, and skeletal components, this review aims to provide a coherent framework for understanding PRDM-mediated regulation in both physiological and pathological contexts, while highlighting current limitations and future research directions.

## 2. Overview of the PRDM Protein Family

An overview of the functions of PRDM family members and their related FOG proteins is provided in Table 1.

PRDM1 (PR domain zinc finger protein 1, also known as Blimp-1) is a key epigenetic regulator associated with terminal T-cell differentiation [6]. PRDM2 (PR domain zinc finger protein 2, PRDM2/RIZ) is characterized by a conserved N-terminal PR domain with histone methyltransferase activity and a C-terminal array of zinc finger motifs [7]. PRDM3 is involved in multiple biological processes, including hematopoiesis, development, cellular differentiation, and apoptosis [8,9]. PRDM4 participates in various aspects of cell differentiation and proliferation [10]. PRDM5 functions as a transcriptional repressor and is considered a putative tumor suppressor in several types of cancer [11]. PRDM6 is a smooth muscle cell (SMC)-specific histone methyltransferase [12]. PRDM7 is a primate-specific histone methyltransferase that shares high sequence homology with PRDM9 within its catalytic PR/SET domain [13]. PRDM8 plays an essential regulatory role in both physiological and pathological processes, including nervous system development, neuronal migration, retinal development, and neurological disorders [14,15]. In addition, PRDM8 is expressed in various tissues, such as the lung [16], testis [17], and skeletal muscle [18].

PRDM9 is an essential enzyme required for progression through early meiotic prophase and plays a crucial role in homologous recombination in most mammals [19]. Alterations in PRDM9—particularly within its zinc finger motifs—are associated with cancer initiation and progression [20], which are characterized by genomic instability and aberrant regulation of genes critical for cell growth, proliferation, and differentiation. PRDM10 (positive regulatory domain I-binding factor 1) is closely associated with the development and progression of various cancers, including nasopharyngeal carcinoma [21] and hepatocellular carcinoma [22], and also plays an important role during embryonic development [23]. PRDM11 is closely associated with defective diffuse large B-cell lymphomas (DLBCLs) and participates in the regulation of key oncogenes, such as FOS and JUN [24]. PRDM12 is expressed in a spatiotemporal manner within the multifunctional nervous system. It was initially identified in chronic myeloid leukemia as a gene located in a deletion region of chromosome 9 and has been shown to regulate the proliferation of various cell lineages. Notably, PRDM12 plays an essential role in initiating and activating neurogenesis by orchestrating the downstream transcriptional cascade in nociceptive neuronal lineages [25]. PRDM13 is crucial for the development of the nervous system and acts as a key regulatory factor in the formation of retinal cells [26], cerebellar GABAergic neurons, and hypothalamic kisspeptin neurons [27]. PRDM14, which contains a single PR domain and six tandem zinc finger motifs, participates in histone deacetylation and methylation processes. It also contributes to tumorigenesis through changes in promoter methylation levels [28]. Aberrant methylation of PRDM14 alters chromatin structure, DNA conformation, and DNA–protein interactions, thereby repressing gene transcription and expression, ultimately promoting tumor initiation, progression, and metastasis [29]. Moreover, PRDM14 plays critical roles in maintaining cellular integrity, differentiation, growth, and apoptosis, particularly in primordial germ cell formation, stem cell pluripotency maintenance, and tissue and organ development [30]. PRDM15 plays a key role in the development of B-cell lymphomas and is involved in the regulation of multiple physiological systems [31]. PRDM16 is essential for maintaining the characteristics and function of brown adipocytes in adult mice. It regulates the differentiation of brown adipocytes and lymphocytes and has been implicated in several types of cancer, including myeloid leukemia [32,33].

Members of the PRDM family exhibit extensive functional associations with the GATA-FOG complex, jointly regulating cell lineage differentiation and tissue development. FOG1 cooperates with the hematopoietic master regulator GATA1 to coordinate the differentiation of platelets and erythrocytes [2,34,35]. FOG1 acts as a co-repressor that links GATA factors to histone deacetylation and nucleosome remodeling machinery [36]. FOG2 functions as a transcriptional regulatory complex for multiple GATA factors and serves as a key determinant of the diversity of corticothalamic projection neurons (CThPNs) [37]. It is essential for the normal function of mesenchymal cells in the developing septum, lungs, and heart [38], and plays a crucial role in cardiac morphogenesis and coronary artery development [39].

**Table 1:** Functions of PRDM family members and related FOG proteins.

Protein	Key Functions	References
PRDM1	Master regulator of terminal T-cell differentiation.	
PRDM2	Histone methyltransferase with PR domain and zinc fingers; regulates transcription and cell fate.	[7,40]
PRDM3	Controls hematopoiesis, development, differentiation, and apoptosis.	[8,9]
PRDM4	Regulates cellular differentiation and proliferation.	[10]
PRDM5	Functions as a transcriptional repressor and potential tumor suppressor.	[11,41]
PRDM6	Smooth muscle-specific histone methyltransferase regulating *SMC* gene programs.	[12]
PRDM7	Primate-specific histone methyltransferase homologous to PRDM9.	[13,42]
PRDM8	Regulates neural development, neuronal migration, and retinal formation.	[5,14,15,43]
PRDM9	Essential enzyme for meiotic progression and homologous recombination; zinc finger variants linked to cancer.	[19,20]
PRDM10	Associated with tumorigenesis and critical for embryonic development.	[21,22]
PRDM11	Implicated in DLBCL; regulates oncogenes such as FOS and JUN.	[24]
PRDM12	Temporally regulated in the nervous system; essential for nociceptor development.	[25]
PRDM13	Key regulator of retinal development and GABAergic neuronal differentiation.	[26,27,44]
PRDM14	Controls histone/DNA methylation; maintains pluripotency and contributes to tumorigenesis.	[28,29,30]
PRDM15	Regulates multiple developmental pathways and contributes to B-cell lymphoma.	[31,45]
PRDM16	Maintains brown adipocyte identity; involved in adipogenesis and hematologic malignancies.	[32,46,47]
FOG1	Cofactor of GATA1, essential for erythroid and megakaryocyte differentiation.	[2,34,35]
FOG2	GATA co-regulator required for cortical–thalamic neuron diversity and cardiac/pulmonary development.	[37,38,39]

## 3. The PRDM Protein Family and the Nervous System

**Figure 3:**
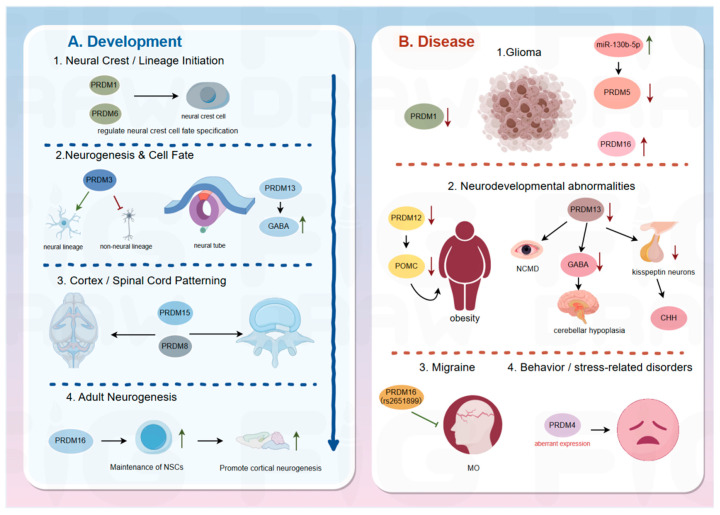
Roles of PRDM family members in neural development and neurological disorders. (**A**) Neural development. PRDM factors participate in multiple stages of neurodevelopment. PRDM1 and PRDM6 regulate neural crest cell fate specification. PRDM3 maintains the balance of neural versus non-neural lineages during early neurogenesis. Within the developing neural tube, PRDM13 governs the differentiation trajectory of GABAergic and glutamatergic neurons. PRDM8 and PRDM15 contribute to cortical and spinal cord patterning, while PRDM16 plays essential roles in several late developmental processes, including the formation of ependymal cells in the SVZ, the maintenance of adult neural stem cells, and adult neurogenesis in the dentate gyrus. (**B**) Neurological diseases. Distinct PRDM members exhibit diverse, and sometimes opposing, functions in pathology. In glioma, several PRDM proteins act as either tumor suppressors or oncogenic regulators. PRDM12 deficiency leads to reduced POMC expression, impaired energy balance, and obesity. PRDM13 is implicated in congenital hypogonadotropic hypogonadism (CHH) and cerebellar hypoplasia, partly through disruption of GABAergic neuron development. PRDM16 is associated with increased susceptibility to migraine, while aberrant expression of PRDM4 has been linked to altered stress-related behaviors and psychological resilience.

In vertebrates, movement is not merely the result of simple muscle contraction but rather a highly orchestrated process governed by complex neural networks. The execution of motor activity relies on the precise control exerted by local spinal circuits, which selectively activate specific motor neurons to drive skeletal muscle fibers and generate coordinated motion. As critical interfaces between the central nervous system and skeletal muscles, motor neurons establish functional connections with muscle fibers, providing the structural basis for precise motor control. In recent years, accumulating evidence has demonstrated that physical exercise can modulate neuromuscular connectivity and synaptic plasticity, thereby influencing motor neuron function and providing new insights into the mechanisms underlying motor regulation. As a family of key epigenetic regulators, PRDM proteins have been implicated in multiple aspects of nervous system development, including motor neuron fate determination and neural circuit formation. Although direct localization of PRDM proteins at the neuromuscular junction (NMJ) has not yet been clearly demonstrated, current evidence suggests that they may indirectly influence NMJ organization and function by regulating transcriptional programs in motor neurons and associated neural circuits (Figure 3). Together, these findings highlight the potential roles of PRDM family members in regulating neuromuscular function.

### 3.1. Regulatory Roles of the PRDM Family in Neuronal Growth and Development

Members of the PRDM family exhibit spatiotemporal specificity and regulatory mechanisms during nervous system development, participating in multiple key processes, including neural crest cell migration, neuronal lineage differentiation, motor neuron fate determination, and the establishment of precise neural circuitry. PRDM1 and PRDM3 play distinct yet equally crucial roles in neurodevelopment. During early embryogenesis, PRDM1 promotes the lineage-specific differentiation of pluripotent cells into neural and sensory lineages by orchestrating the sequential activation of transcriptional programs governing neural, neural crest, and sensory progenitor cells, thereby serving as a pivotal determinant of neural crest cell fate [48]. In adulthood, PRDM1 remains expressed in the dorsal root ganglia (DRG), contributing to the maintenance of peripheral sensory neuron function. Under conditions of neural injury, PRDM1 modulates nociceptive responses by downregulating Kv4.3 potassium channel expression, suggesting its critical role in the adaptive response of the peripheral nervous system to stress [49].

In contrast, PRDM3 exerts its regulatory functions during neurogenesis primarily through transcriptional induction mechanisms. Loss of PRDM3 leads to premature neuronal differentiation in P19 cells, accompanied by enhanced proliferation of non-neuronal lineage cells, underscoring its critical role in maintaining neural lineage balance and suppressing alternative, non-neuronal fates [50].

As an epigenetic modifier, PRDM6 plays a central role in regulating neural crest cell fate determination. Through spatiotemporally specific expression, PRDM6 governs lineage specification within neural crest populations—particularly in cardiac neural crest cells, where it determines differentiation toward distinct cell types. As a key regulatory factor within the neural crest cell-fate-determining network, PRDM6 is considered a potential therapeutic target for modulating the development and progression of congenital heart diseases [51].

PRDM8 exhibits spatiotemporally specific expression during nervous system development and participates in neuronal and glial fate determination through multiple regulatory mechanisms. In the early stages of neurodevelopment, PRDM8 is predominantly expressed in postmitotic neurons, with particularly strong expression observed in the intermediate zone and cortical plate of the developing cerebral cortex. As cortical maturation proceeds, *PRDM8* expression becomes progressively restricted to layer IV neurons of the neocortex, where it plays a critical role in the proper construction of upper-layer (UL) cortical neurons in mammals [16,52,53]. In the spinal cord, *PRDM8* expression initially appears in progenitor domains of ventral interneurons and motor neurons, and later expands to multiple interneuron subtypes. Evidence suggests that PRDM8 regulates Sonic hedgehog (Shh) signaling activity in pMN progenitor cells, thereby mediating the lineage switch between motor neurons and oligodendrocyte progenitor cells (OPCs). This indicates its pivotal role in determining the lineage allocation between motor neurons and myelination-associated cells [52]. Moreover, PRDM8 forms a transcriptional repressor complex with the basic helix–loop–helix (bHLH) transcription factor Bhlhb5 (also known as Bhlhe22) to regulate target gene expression. For example, by precisely modulating the expression of Cadherin-11, PRDM8 contributes to the fine assembly of neuronal circuits, ensuring accurate synaptic connectivity during cortical development [53].

PRDM12 is a key epigenetic regulator involved in the development of sensory neurons and the expression of nociception-related genes, functioning across multiple stages of neurogenesis. During embryonic development, PRDM12 is essential for initiating the neural lineage transcriptional program, particularly for specifying nociceptor progenitors. By repressing genes associated with non-nociceptive lineages, PRDM12 directs progenitor cells toward specific nociceptor subtypes, thereby playing a crucial role in cell lineage determination within the sensory nervous system. In both Xenopus embryos and human fetuses, *PRDM12* is highly expressed in nociceptors and their progenitors, indicating its strong evolutionary conservation among vertebrates [54]. In adults, PRDM12 exerts complex regulatory functions in nociceptive modulation by controlling the expression of specific genes, such as those encoding sodium channels and neuropeptides, and the excitability of sensory neurons, ultimately influencing pain perception behaviors [55]. In the P19 embryonal carcinoma cell differentiation model, PRDM12 participates in the fate determination of inhibitory neurons and astrocytes by regulating inhibitory neuron-specific genes (e.g., *Gad1/2*, *Glyt2*) and their upstream transcription factors (Ptf1a, Dbx1, Gsx1/2, Foxa1, Hic1), thereby governing inhibitory neuronal lineage formation [56].

Moreover, PRDM12 expression levels influence cellular proliferative states: its overexpression suppresses cell cycle progression (evidenced by an increased proportion of G1-phase cells and upregulation of the cell cycle inhibitor p27), whereas its loss promotes retinoic acid-induced neuronal differentiation, suggesting a role for PRDM12 in maintaining progenitor cell homeostasis [25].

PRDM13 regulates the balance between GABAergic and glutamatergic neuronal fates in the dorsal and caudal regions of the vertebrate neural tube [57,58]. During neural tube development, PRDM13 maintains the lineage equilibrium between GABAergic and glutamatergic neurons by recruiting various basic helix–loop–helix (bHLH) transcription factors to chromatin, thereby restricting gene expression in specific neuronal lineages. It represses the transcriptional programs associated with excitatory neuronal lineages in the dorsal neural tube. In summary, PRDM13 primarily exerts its function by inhibiting the activity of bHLH transcriptional activators [59]. Furthermore, PRDM13 plays an essential role in determining retinal neuronal subtypes. It is highly expressed in the fetal retina, particularly within the neurite-less precursor cell population, and acts as a key determinant of the generation of Ebf3^+^ glycinergic neuronal subtypes [44,60]. The expression of PRDM13 is subject to negative feedback regulation, and its overexpression promotes the differentiation of glycinergic neurite-less neurons, suggesting that PRDM13 may help maintain retinal circuit stability by modulating the proportion of inhibitory neurons.

PRDM15 is an important transcriptional regulator that maintains the pluripotent state of embryonic stem cells and has recently been shown to play a pivotal role during early neurodevelopment. By modulating the expression of upstream signaling components such as Rspo1 and Spry1, PRDM15 activates the WNT and MAPK-ERK pathways, thereby stabilizing the pluripotency transcriptional network and preventing premature differentiation of embryonic stem cells into the neural lineage [61]. Loss of PRDM15 results in embryonic developmental defects, accompanied by a marked downregulation of core genes in several key signaling pathways, including WNT, NOTCH, and SHH. These findings suggest that PRDM15 modulates the activity of these pathways through chromatin-state regulation, thereby determining the timing and magnitude of neural lineage initiation [61]. In non-mammalian species such as Xenopus laevis, PRDM15 exhibits a conserved function. Knockdown of PRDM15 significantly suppresses the expression of neurodevelopmental regulators such as pax6 and otx2, leading to impaired formation of the forebrain, optic vesicles, and neural crest cells [62]. Collectively, these observations highlight PRDM15 as a central regulatory factor controlling neural lineage establishment and regional patterning during early neurodevelopment.

The functional significance of PRDM16 in multiple key processes of nervous system development has been well established. It plays a critical role in the formation of ependymal cells lining the subventricular zone (SVZ) and in maintaining neural stem cells and neurogenesis within the dentate gyrus (DG) of the adult brain [63]. During early development, PRDM16 is essential for guiding sympathetic axon projections and is closely associated with the initial growth of sympathetic neurons. Rather than binding directly to DNA, PRDM16 exerts its regulatory function by forming complexes with specific interacting partners. In the developing nervous system, PRDM16 associates with C/EBPβ to modulate gene expression—a mechanism that also underlies its role in promoting brown adipose tissue differentiation [64]. Selective ablation of PRDM16 in RGPs leads to elevated levels of reactive oxygen species (ROS) and induces the expression of oxidative stress-responsive genes, indicating that PRDM16 is indispensable for preserving the homeostatic environment necessary for proper neurogenesis.

### 3.2. The Involvement of the PRDM Family in the Pathogenesis of Neurological Diseases

Members of the PRDM family play crucial regulatory roles in the onset and progression of various neurological disorders. PRDM1 has been identified as a tumor suppressor in multiple cancers. In gliomas and lower-grade gliomas (LGG), its expression level has been associated with tumor stage and the characteristics of the immune microenvironment [65]. However, direct evidence of a negative correlation between PRDM1 protein expression and WHO pathological grade in gliomas remains lacking. Likewise, whether restoration of *PRDM1* expression can significantly suppress glioma cell proliferation and invasion remains to be clarified. In addition, loss of PRDM1 has been shown to accelerate the onset and progression of melanoma in zebrafish models, where prdm1a deficiency impairs neural crest cell differentiation into melanocytes. Consistently, reduced PRDM1 expression is associated with poorer survival outcomes in melanoma patients [66], suggesting a protective role of PRDM1 in tumorigenesis.

A large-scale human study involving 9120 participants demonstrated that aberrant PRDM4 expression is associated with a reduced psychological endurance threshold (PET) and an increased propensity for abnormal behavioral responses. Individuals with lower PET values were more likely to exhibit maladaptive or extreme behavioral outcomes [67], implying a potential involvement of PRDM4 in the regulation of psychiatric or behavioral disorders.

PRDM5 also exhibits tumor-suppressive properties in the nervous system. Its expression is significantly downregulated in glioma tissues and correlates with poor clinical outcomes in patients [68]. Mechanistically, PRDM5 has been identified as a direct target of miR-130b-5p. In acute spinal cord injury, the lncRNA BDNF-AS alleviates neuronal apoptosis by downregulating miR-130b-5p, thereby releasing its inhibition on PRDM5. Similarly, PRDM5 is negatively regulated by miR-495; overexpression of miR-495 reduces hypoxia-induced apoptosis in PC-12 cells, while PRDM5 overexpression reverses this protective effect [69]. Furthermore, PRDM5 suppression facilitates cell proliferation by modulating cell cycle progression, and siRNA-mediated knockdown of PRDM5 decreases apoptosis in glioma cells, suggesting that PRDM5 may serve as a novel therapeutic target for glioma treatment.

Biallelic autosomal recessive mutations in PRDM12 have been identified as the genetic cause of Middle Face Toddler Excoriation Syndrome (MiTES) [70]. In addition, PRDM12 is involved in establishing the fate of hypothalamic pro-opiomelanocortin (POMC) neurons. Loss of PRDM12 impairs Pomc expression, leading to increased food intake and obesity [71], and it has also been implicated in the regulation of anorexia-associated neurons and body weight homeostasis [72].

PRDM13 acts as a key regulator of cerebellar GABAergic neurons and hypothalamic kisspeptin neurons development, providing a mechanistic explanation for congenital hypogonadotropic hypogonadism (CHH) and cerebellar hypoplasia [27]. Loss of PRDM13 causes a significant reduction in GABAergic and glycinergic neurites, resulting in specific defects in the S2/S3 marginal plexiform bundles of the inner plexiform layer. In zebrafish, loss of PRDM13 function leads to a reduced number of Purkinje cells and a complete absence of the inferior olive nucleus, further demonstrating that biallelic PRDM13 mutations can cause olivopontocerebellar hypoplasia syndrome. Moreover, North Carolina macular dystrophy (NCMD) has been found to contain tandem repeat sequences within the *PRDM13* gene [73].

PRDM16 (rs2651899) has been identified as a potential genetic marker associated with migraine susceptibility [74]. The rs2651899 variant genotype and allele of PRDM16 are considered protective against migraine and migraine without aura (MO) [75]. Further studies have revealed that rs2651899, located on chromosome 1p36.32 within the *PRDM16* gene, is a potential genetic marker for migraine susceptibility, particularly in MO and in the female subgroup, among North Indian populations. In the Chinese population, genome-wide association studies (GWAS) have similarly shown a significant association between PRDM16 and migraine susceptibility, especially MO [76]. Notably, PRDM16 is also highly expressed in atypical teratoid/rhabdoid tumors (ATRT), and its knockout markedly suppresses the proliferation of SMARCB1-deficient cells [77].

## 4. PRDM Family and the Musculoskeletal System

### 4.1. The Involvement of the PRDM Family in Skeletal Cell Development

During physical activity, the skeleton functions not only as a structural “framework” but also as a dynamic, adaptive “living tissue”. Mechanical loading and gravitational stimuli activate osteoblasts, osteoclasts, and bone progenitor cells, promoting bone remodeling that optimizes bone density and structure, thereby enhancing compressive strength and resistance to fracture [78]. Exercise regulates bone cell function not only through mechanical stimulation but also via epigenetic mechanisms that exert long-lasting effects on bone development and remodeling. Epigenetic modifications, such as DNA methylation, histone acetylation, and deacetylation, play critical roles in the differentiation and functional regulation of osteoblasts and osteoclasts during bone remodeling [79]. Members of the PRDM family are crucial regulators of skeletal development. By precisely controlling the differentiation and activity of osteoblasts, osteoclasts, and chondrocytes, PRDM proteins contribute to bone growth, repair, and remodeling. As epigenetic regulators, PRDM members maintain skeletal homeostasis by modulating the lineage specification of bone-related cells and serving as key mediators in the dynamic balance of bone metabolism.

PRDM1, a transcriptional repressor, plays a pivotal role in skeletal development, particularly in osteoblast and osteoclast differentiation and function. Conditional inactivation of PRDM1 has been shown to suppress osteoclast formation in both male and female adult mice, resulting in increased bone mass, suggesting that PRDM1 may serve as a potential therapeutic target to enhance bone density and inhibit osteoclastogenesis [80]. Conversely, overexpression of PRDM1 promotes osteoclast formation [81]. During RANKL-mediated osteoclast differentiation, PRDM1 suppresses the expression of Bcl6. Loss of PRDM1 impairs Bcl6 downregulation, leading to persistent inhibition of osteoclastogenic genes, which, in turn, severely suppresses osteoclast formation and increases bone mass [82]. Moreover, the PR domain-containing zinc finger protein PRDM1 also exhibits pro-inflammatory properties. Since inflammation is a potent driver of osteoclast differentiation and a key process in the pathogenesis of acute gouty arthritis, PRDM1 may participate in the inflammatory regulation of bone-resorptive processes.

PRDM2, another critical member of the PR domain family, plays essential roles in cell cycle regulation, differentiation, and apoptosis. It is expressed in both immature progenitors and mature bone marrow cells, indicating its involvement in multiple differentiation stages within the bone marrow microenvironment [83]. During RANKL-induced osteoclast precursor differentiation, *PRDM2* expression is markedly upregulated. Silencing PRDM2 suppresses the expression of the key transcription factor nuclear factor of activated T cells 1 (NFATc1), thereby delaying osteoclast formation and maturation. NFATc1 functions as a master transcriptional regulator of osteoclastogenesis by directly activating a panel of osteoclast-specific genes, including cathepsin K (Ctsk), matrix metalloproteinase-9 (MMP-9), osteoclast-associated receptor (OSCAR), dendritic cell-specific transmembrane protein (DC-STAMP), and Atp6v0d2 [84,85]. These genes are essential for osteoclast differentiation, multinucleation, and bone-resorptive activity, thereby establishing NFATc1 as a central effector downstream of PRDM2 signaling. Mechanistically, PRDM2 may influence NFATc1 transcriptional activity by modulating classical signaling pathways, such as NF-κB and MAPK, thereby participating in RANKL-mediated osteoclastogenesis [81].

In addition, the splicing variant of PRDM2, known as RIZ1, has been demonstrated to play a regulatory role in myeloid cell differentiation and is closely associated with the onset and progression of myelodysplastic syndromes (MDS) [83,86]. In RAW264.7 murine macrophage-like cells, *RIZ1* expression is markedly upregulated following RANKL stimulation, whereas its silencing significantly suppresses the expression of *Trap* and *Nfatc1*, suggesting that *RIZ1* may facilitate osteoclast differentiation by modulating *Nfatc1* transcriptional activity [87].

PRDM3 is aberrantly expressed in approximately 10% of bone marrow samples from MDS patients. This disorder is characterized by severe anemia and multilineage myeloid dysplasia, which represent major pathological features contributing to mortality in MDS [88].

Furthermore, PRDM16 has been shown to promote chondrocyte differentiation and modulate osteoblast maturation, possibly by transiently repressing *Runx2* expression during the early stages of osteo-chondrogenic commitment, thereby favoring chondrogenic lineage specification [89,90]. Genetic association studies have identified polymorphisms in PRDM16 that are significantly correlated with whole-body bone mineral density (BMD) in postmenopausal Japanese women, indicating its potential involvement in bone metabolic regulation [72]. PRDM16 is abundantly expressed in the pharyngeal arches, and its loss results in the downregulation of neural crest cell markers (dlx2a and barx1), leading to defects in viscerocranial and cranial morphogenesis as well as aberrant craniofacial development in zebrafish [91]. Moreover, PRDM16 participates in the differentiation of mesenchymal stem cells into chondrocytic and osteogenic lineages, and its deficiency causes complete clefting of the secondary palate, accompanied by a significant reduction in H3K9me3 methylation levels within the palatal shelves [92]. Collectively, PRDM3 and PRDM16 exert pivotal roles in craniofacial development by orchestrating the spatiotemporal gene regulatory networks necessary for the proper development of cranial neural crest cells (cNCCs). These regulatory mechanisms are evolutionarily conserved among vertebrates, while also exhibiting species-specific divergence [93].

### 4.2. The Role of the PRDM Family in Skeletal Muscle Growth and Development

As the central effector component of the musculoskeletal system, skeletal muscle not only drives body movement but also plays pivotal roles in energy metabolism, thermogenesis, and mechanotransduction. Exercise training markedly promotes skeletal muscle growth, regeneration, and fiber-type remodeling—a process that critically depends on the activation and differentiation of muscle stem cells (satellite cells) and is tightly governed by intricate transcriptional and epigenetic regulatory networks [94].

The formation and differentiation of skeletal muscle constitute a highly coordinated, multistep process involving cell proliferation, lineage commitment, fusion, and terminal differentiation, all of which are strictly controlled by diverse epigenetic regulators [95]. Although research on the PRDM family in skeletal muscle development remains relatively limited compared with that in the nervous or hematopoietic systems, current evidence indicates that PRDM members play essential roles in myogenic lineage specification, regulation of myogenic differentiation programs, and stem/progenitor cell reprogramming, highlighting their potential as key modulators in muscle growth and regeneration.

In vertebrates, skeletal muscle is composed of distinct fiber types that are functionally specialized according to their physiological and metabolic characteristics [96]. Type I fibers, also known as slow-twitch fibers, contract at a slower rate and are rich in mitochondria, conferring a high oxidative capacity. These fibers efficiently utilize oxygen to generate ATP, thereby enhancing muscle endurance [96]. In contrast, Type II fibers (fast-twitch fibers) are optimized for rapid, high-intensity contractions over short durations. Owing to their fast contractile speed and strong glycolytic capacity, they fatigue more rapidly [97].

PRDM1 is a key epigenetic regulator of muscle fiber fate, playing a crucial role in the formation of slow muscle fibers, particularly in zebrafish. PRDM1 modulates slow muscle differentiation through a dual mechanism: acting as a global repressor of fast muscle-specific genes while simultaneously relieving transcriptional repression of slow muscle genes [98]. Although typical slow muscle markers such as smyhc1, tnnc1b, and prox1 have not been directly identified among PRDM1a targets, their regulatory effects are likely mediated by suppressing transcriptional repressors (e.g., Sox6), thereby indirectly promoting slow fiber specification [99]. The *PRDM1* gene is highly conserved across vertebrates, yet its regulatory roles exhibit species-specific diversification. For instance, in mice, its expression within somites depends on Hedgehog (Hh) signaling domains, although myotome formation itself is not strictly dependent on PRDM1 activity [100]. In chickens, *PRDM1* expression is neither exclusive to fast nor slow fibers, and it is detectable during both early myogenesis and terminal differentiation [101]. Collectively, these findings indicate that PRDM1-mediated regulation of muscle fiber identity demonstrates both evolutionary conservation and functional plasticity.

PRDM2 primarily maintains the quiescence of undifferentiated myogenic cells. By binding to the promoters of key myogenic regulators such as MyoG, PRDM2 suppresses differentiation during the G_0_ phase, preserving the undifferentiated state of myogenic progenitors. Knockdown of PRDM2 significantly alters the histone methylation pattern at the promoters of MyoG and CyclinA2, thereby relieving dual repression of myogenic differentiation and cell cycle progression. This disruption leads to a loss of cellular quiescence and premature initiation of myogenesis [40]. These observations underscore PRDM2 as a critical determinant of the temporal window governing myogenic cell fate decisions.

PRDM16 serves as a pivotal epigenetic modulator at the crossroads between brown adipocyte and skeletal muscle lineage determination. In C2C12 myoblasts, ectopic expression of PRDM16 induces transdifferentiation into brown fat-like cells, accompanied by hypermethylation of the MyoG and MyoD promoters and activation of lipid-metabolism-related genes such as PPARγ [46,102]. Moreover, the myogenic regulatory factors Myf5 and MyoD suppress *PRDM16* expression by activating the E2F4/p107/p130 transcriptional repressor complex, thereby preventing this lineage conversion [102]. This mechanism highlights PRDM16’s central role in balancing lineage stability and plasticity within skeletal muscle progenitors.

In summary, members of the PRDM family exert multifaceted regulatory functions throughout the various stages of skeletal muscle growth and development, integrating transcriptional and epigenetic cues to coordinate lineage specification, differentiation, and adaptive remodeling.

## 5. Conclusions and Perspectives

The PRDM family comprises pivotal epigenetic regulators that play essential roles in cell fate determination, tissue development, and disease pathogenesis across the neuro-motor system. Accumulating evidence indicates that PRDM proteins function as transcriptional and chromatin modulators, integrating genetic and environmental cues to regulate neuronal differentiation, neural circuit formation, skeletal homeostasis, and myogenic processes. These findings highlight the broad biological relevance of PRDM family members in coordinating neuro-musculoskeletal interactions, yet current knowledge is disproportionately focused on a limited subset of well-characterized proteins, leaving many members functionally underexplored.

However, current understanding of PRDM family members, particularly in neurological diseases, remains limited and uneven. Most available evidence is derived from high-throughput transcriptomic and epigenomic analyses, providing predominantly correlative insights rather than mechanistic or causal validation. While PRDM functions in developmental contexts have been relatively well characterized, their roles in disease progression—especially in neurological disorders—remain largely descriptive. In addition, evidence linking PRDM proteins to specific muscle diseases, such as muscular dystrophy, is still sparse and lacks disease subtype specificity, with most conclusions extrapolated from general myogenic regulatory mechanisms.

Several key gaps should be addressed. First, the causal roles of PRDM dysregulation in disease remain unclear. Second, the functional diversity of PRDM isoforms generated through alternative promoter usage has not been systematically investigated, despite potential opposing effects. Third, the involvement of PRDM family members in neuro-muscular interactions, particularly at the neuromuscular junction, remains largely unexplored. These limitations reflect a broader gap between large-scale omics-based discoveries and mechanistic validation.

Future studies should focus on bridging the gap between descriptive and mechanistic research by integrating single-cell multi-omics, chromatin mapping, and functional genomic approaches. In particular, isoform-specific analyses and tissue-specific models will be essential to clarify the context-dependent roles of PRDM proteins. Elucidating their functions in neuro-muscular interactions may further advance our understanding of system-level regulation and facilitate the development of more precise therapeutic strategies.

## Data Availability

Data availability is not applicable to this review article, as it is based exclusively on previously published studies and does not include any original data.
